# Intramedullary spinal cord metastasis from colonic carcinoma presenting as Brown-Séquard syndrome: a case report

**DOI:** 10.1186/1752-1947-5-342

**Published:** 2011-08-02

**Authors:** Mohammed A Kaballo, Darren D Brennan, Mazen El Bassiouni, Stephen J Skehan, Rajnish K Gupta

**Affiliations:** 1 Mid-Western Cancer Centre, Mid-Western Regional Hospital, Dooradoyle, Limerick, Ireland; 2 Department of Radiology, Mid-Western Regional Hospital, Dooradoyle, Limerick, Ireland; 3 Department of Radiation Oncology, Mid-Western Regional Hospital, Dooradoyle, Limerick, Ireland; 4 Department of Nuclear Medicine, Blackrock Clinic, Dublin, Ireland; 5 Stokes Institute, University of Limerick, Ireland

## Abstract

**Introduction:**

Intramedullary spinal cord metastasis is very rare. The majority are discovered incidentally during autopsy. Most symptomatic patients present with rapidly progressive neurological deficits and require immediate examination. Few patients demonstrate features of Brown-Séquard syndrome. Radiotherapy is the gold-standard of therapy for Intramedullary spinal cord metastasis. The overall prognosis is poor and the mortality rate is very high. We present what is, to the best of our knowledge, the first case of Intramedullary spinal cord metastasis of colorectal carcinoma presenting as Brown-Séquard syndrome.

**Case presentation:**

We present the case of a 71-year-old Caucasian man with colonic adenocarcinoma who developed Intramedullary spinal cord metastasis and showed features of Brown-Séquard syndrome, which is an uncommon presentation of Intramedullary spinal cord metastasis.

**Conclusion:**

This patient had an Intramedullary spinal cord metastasis, a rare form of metastatic disease, secondary to colonic carcinoma. The metastasis manifested clinically as Brown-Séquard syndrome, itself a very uncommon condition. This syndrome is rarely caused by intramedullary tumors. This unique case has particular interest in medicine, especially for the specialties of medical, surgical and radiation oncology. We hope that it will add more information to the literature about these entities.

## Introduction

Intramedullary spinal cord metastasis (ISCM) is very rare, accounting for only 0.9-5.0% of all spinal cord metastases. The majority are discovered incidentally during autopsy, and manifest clinically in only 0.1-0.4% of cancer patients. Only 3% of pathologically confirmed ISCM are secondary to colorectal cancer. Most symptomatic patients present with rapidly progressive neurological deficits and require immediate examination. Of these, 23% demonstrate features of Brown-Séquard syndrome. Radiotherapy is the gold-standard of therapy for ISCM. The overall prognosis is poor; mortality rate is 80% during the first three to four months after the appearance of the first symptom. We present what is, to the best of our knowledge, the first case of an ISCM of colorectal carcinoma presenting as Brown- Séquard syndrome.

## Case presentation

A 71-year-old Caucasian man was diagnosed with poorly differentiated pT3N2M0 adenocarcinoma of the transverse colon. He underwent a transverse colectomy followed by adjuvant chemotherapy which he could not tolerate, and therefore stopped after three cycles. One year later he developed a single metastasis to the left lobe of his liver which was successfully resected. Following the resection he tolerated 12 cycles of adjuvant chemotherapy (irinotecan and de Gramont) and continued in remission.

**Figure 1:**
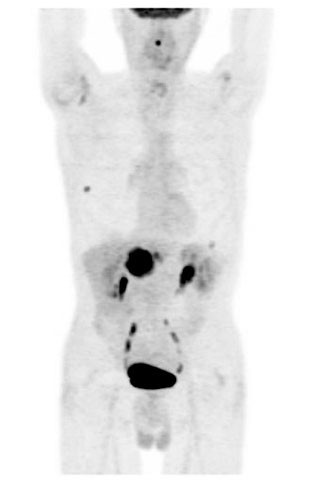
**FDG PET-CT maximum intensity projection image showing three FDG-avid pulmonary nodules**.

**Figure 2:**
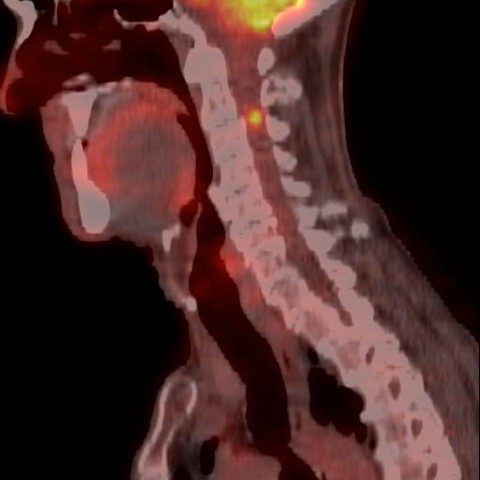
**Sagittal fused PET-CT scan of his cervical spine showing a small focus of intense tracer uptake in the spinal canal at C2-C3 level in his neck**.

**Figure 3:**
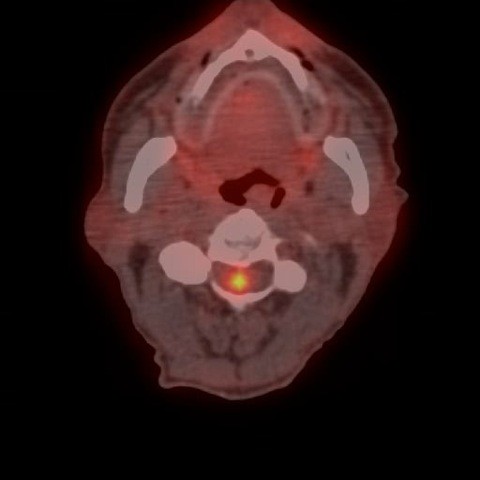
**Axial fused PET-CT scan of the cervical spine showing a small focus of intense tracer uptake in the spinal canal at C2-C3 level in the neck**.

Four months after finishing chemotherapy, a surveillance computed tomography (CT) scan showed a new single liver nodule highly suspicious of metastasis. Another surgical resection was being considered and a positron emission tomography (PET) CT scan was performed to exclude the presence of metastases elsewhere. Unfortunately, three fluorine-18-fluorodeoxyglucose (FDG) -avid pulmonary nodules were found, as well as a small focus of intense tracer uptake in the spinal canal at the C2-C3 level in his neck (Figures 1, 2, 3). These findings precluded surgical resection. At that time our patient was totally asymptomatic from a neurological point of view.

One week later our patient started to feel pins and needles in his right upper and lower limbs. His symptoms worsened rapidly over the next three days and progressed to motor weakness. Two days later he was unable to walk without using a stick or being supported. There was no history of trauma and he denied any loss of sphincteric control. He was referred and reviewed as an emergency and admitted on the same day. A neurological examination revealed the following signs on his right side: spasticity, hyperreflexia (all right deep tendon reflexes), weakness, decreased tactile discrimination, joint and vibration sense, and upgoing plantar reflex. On his left side, the only abnormality was decreased temperature and pain sensations below the C5 dermatome level. There were no extrapyramidal signs on either side. His coordination was normal.

**Figure 4:**
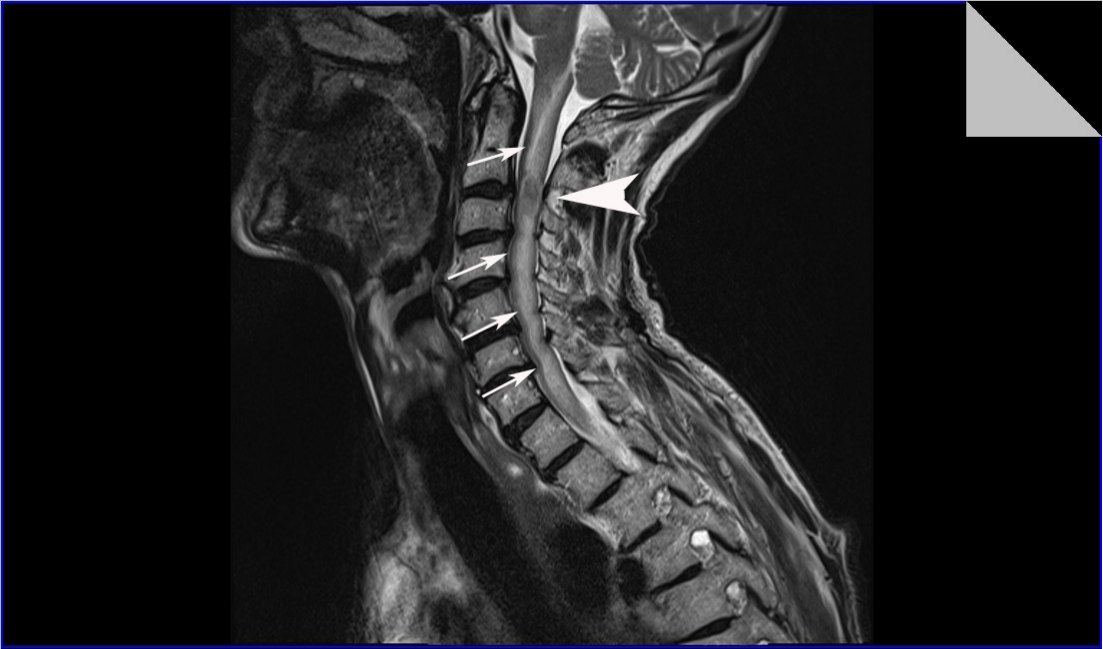
**Sagittal T2-weighted MRI of his cervical spine shows an oblong expansile intramedullary lesion at the C2-C3 level of his spinal cord, associated with large proximal and distal syrinx and edema extending from the cervico-medullary junction to the inferior aspect of the T3 vertebral body**.

**Figure 5:**
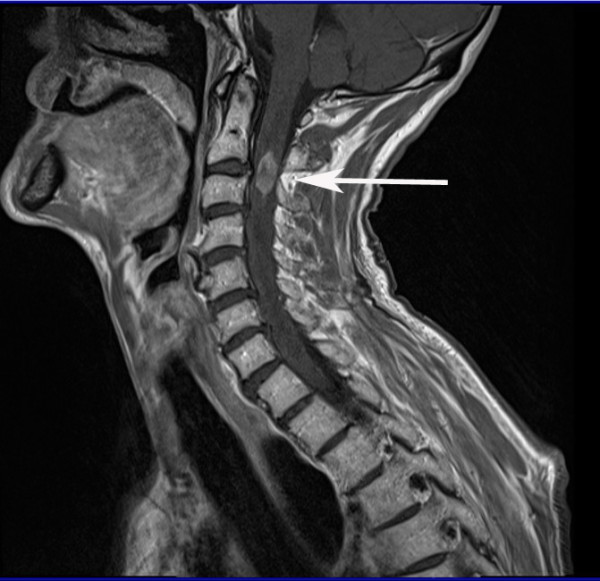
**Sagittal T1-weighted MRI of his cervical spine**.

**Figure 6:**
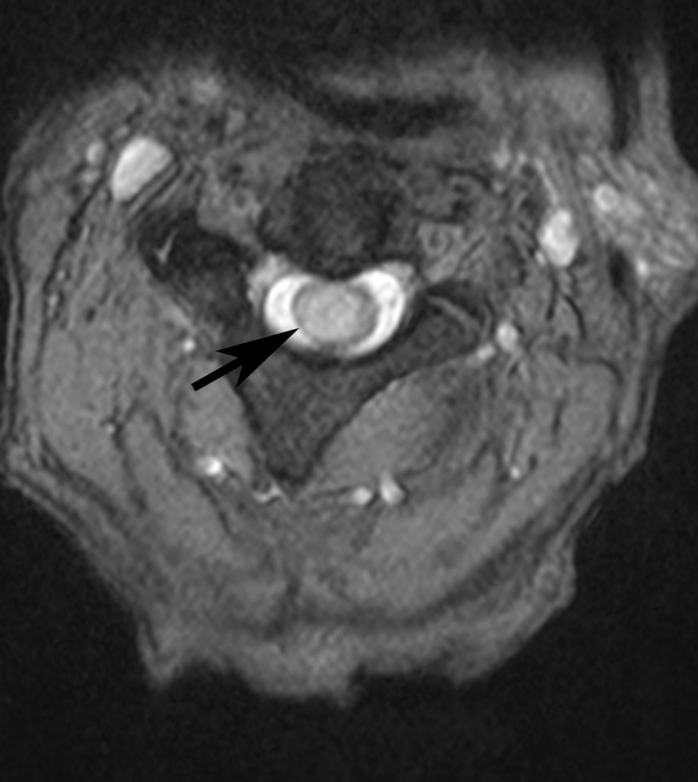
**Axial T2-weighted MRI of his cervical spine showed that the lesion is slightly to the right of the midline**.

Our patient was immediately started on intravenous high-dose dexamethasone and an urgent magnetic resonance imaging (MRI) of his cervical spine was performed that day. The scan showed an oblong expansile intramedullary lesion at the C2/C3 level of the spinal cord, associated with a large proximal and distal syrinx and edema extending from the cervico-medullary junction to the inferior aspect of his T3 vertebral body. The lesion enhanced post-contrast and measured about 2 cm in cranio-caudal dimension. It was slightly to the right of the midline. There was no evidence of bone involvement (Figure 4, 5, 6).

Surgical resection of this intramedullary lesion was not an option in the presence of other lesions in the liver and the lungs, and therefore our patient was referred for palliative radiotherapy.

## Discussion

Spinal cord tumors are either extramedullary or intramedullary. The latter are less common and they form only 5-10% of cases, a minority of which are due to metastases. Of 1096 carcinoma patients studied prospectively at autopsy by Chason *et al*., metastases to the central nervous system (CNS) were found in 200. Only 10 (5%) of these 200 patients had ISCM. If one considers the entire patient population, less than 1% of their cancer patients developed metastases to the spinal cord [1,2]. This low incidence may be attributed to the fact that the spinal cord is not a site that is examined frequently during routine autopsy [3]. But in general, for reasons unknown, the likelihood of malignant tumors metastasizing to the cord intramedullary is low. Lung cancer was found to be the most common source of ISCM, accounting for 64% of the reported cases, followed by breast cancer with 11%, melanoma 5%, renal cell cancer 4%, colorectal cancer 3%, and lymphoma 3%; the primary tumor remained unidentified in 5% of cases [4-10].

Brown-Séquard syndrome is a rare syndrome and most commonly described in conjunction with a traumatic injury to the spinal cord [11]. It is rarely caused by tumors. Brown-Séquard syndrome was found in 23% of patients with ISCM [12-14]. This syndrome is defined as an incomplete lesion of the spinal cord characterized by ipsilateral upper motor neuron paralysis and loss of proprioception, with contralateral loss of pain and temperature sensation. A zone of partial preservation or segmental ipsilateral lower motor neuron weakness and analgesia may be noted. Loss of ipsilateral autonomic function can result in Horner's syndrome. As an incomplete spinal cord syndrome, the clinical presentation of Brown-Séquard syndrome may range from mild to severe neurologic deficit [15,16]. Generally, it is uncommon to find the full picture of Brown-Séquard syndrome, hence it often being called incomplete or partial.

There are two major features to differentiate clinically between primary spinal tumors and ISCM. Acute presentation with rapidly worsening symptoms and the presence of metastases in other organs are typical of ISCM. MRI is highly sensitive for detecting ISCM and demonstrating the edema surrounding them [12]. PET has a sensitivity of 96% in detecting spinal metastasis. This is even more sensitive when combined with a CT scan [17]. Radiotherapy is the gold-standard of therapy for ISCM with long lasting remissions described in some patients with lymphoma and small cell lung cancer [18,19]. Steroids can be given to reduce edema, offering clinical improvement without survival benefits. Chemotherapy has failed to show any survival benefits, as described in some limited studies [8,12]. Microsurgical resection is a treatment option but not appropriate in most patients, who will often have other limiting co-morbidities. Surgery is limited by the patient's age, performance status, location and severity of the primary neoplasm, presence of other metastases and biologic characteristics of the tumor [12,13].

The overall prognosis of ISCM is poor; mortality rate is 80% during the first three to four months after the appearance of the first symptom. The outcome is worse with poorly differentiated tumors and those from lung primaries [6,13,20].

## Conclusion

Our patient had a rare diagnosis - an ISCM (a rare form of metastatic disease) secondary to colonic carcinoma, resulting in a presentation with a rare manifestation--Brown-Séquard syndrome, which itself a very uncommon condition. It is uncommon for this syndrome to be caused by intramedullary tumors.

We hope that this report will add more information to the literature about these entities.

## Consent

Written informed consent was obtained from the patient for publication of this case report and any accompanying images. A copy of the written consent is available for review by the Editor-in-Chief of this journal.

## Competing interests

The authors declare that they have no competing interests.

## Authors' contributions

MAK was the major contributor in studying the case and writing the manuscript and was involved in the medical care of the patient. DDB and SJS were the radiologists who performed the imaging and analyzed the data. MEB was the radiation oncologist responsible for radiation therapy of this patient. RKG is the head of the department and the medical oncologist responsible for the medical care of the patient. All authors read and approved the final manuscript.

## References

[B1] Chason JL Walker FB Landers JW Metastatic carcinoma in the central nervous system and dorsal root ganglia. A prospective autopsy study Cancer 1963 16 781 787 10.1002/1097-0142(196306)16:6<781::AID-CNCR2820160614>3.0.CO;2-M 14020314

[B2] Post MJ Quencer RM Green BA Montalvo BM Tobias JA Sowers JJ Levin IH Intramedullary spinal cord metastases, mainly of nonneurogenic origin AJR Am J Roentgenol 1987 148 5 1015 1022 3554916 10.2214/ajr.148.5.1015

[B3] Amin R Intramedullary spinal metastasis from carcinoma of the cervix. A case report The British Journal of Radiology 1999 72 853 89 91 10341697 10.1259/bjr.72.853.10341697

[B4] Costigan DA Winkelman MD Intramedullary spinal cord metastasis. A clinicopathological study of 13 cases J Neurosurg 1985 62 2 227 233 10.3171/jns.1985.62.2.0227 3968561

[B5] Kaya RA Dalkiliç T Ozer F Aydin Y Intramedullary spinal cord metastasis: a rare and devastating complication of cancer - two case reports Neurol Med Chir (Tokyo) 2003 43 12 612 615 10.2176/nmc.43.612 14723269

[B6] Grem JL Burgess J Trump D Clinical features and natural history of intramedullary spinal cord metastasis Cancer 1985 56 9 2305 2314 10.1002/1097-0142(19851101)56:9<2305::AID-CNCR2820560928>3.0.CO;2-X 4052974

[B7] Edelson RN Deck MD Posner JB Intramedullary spinal cord metastases. Clinical and radiographic findings in nine cases Neurology 1972 22 12 1222 1231 4346871 10.1212/wnl.22.12.1222

[B8] Holoye P Libnoch J Cox J Kun L Byhardt R Almagro U McClelland S Chintapali K Spinal cord metastasis in small cell carcinoma of the lung Int J Radiat Oncol Biol Phys 1984 10 3 349 356 10.1016/0360-3016(84)90053-1 6323364

[B9] Jellinger K Kothbauer P Sunder-Plassmann E Weiss R Intramedullary spinal cord metastases J Neurol 1979 220 1 31 41 10.1007/BF00313146 84065

[B10] Komaki R Cox JD Holoye PY Byhardt RW Changes in the relative risk and sites of central nervous system metastases with effective chemotherapy and radiation therapy for small cell carcinoma of the lung Am J Clin Oncol 1983 6 5 515 521 6310985

[B11] Sinha MK Malhotra HS Agarwal V Garg RK Kar AM Brown-Séquard's syndrome produced by hemicord myelitis - a case report Ann Neurosciences 2008 15 1 25 26 10.5214/ans.0972.7531.2008.150105

[B12] Hrabalek L Intramedullary spinal cord metastasis: review of the literature Biomed Pap Med Fac Univ Palacky Olomouc Czech Repub 2010 154 2 117 122 20668492 10.5507/bp.2010.018

[B13] Kalayci M Cagavi F Gul S Yenidunya S Acikgoz B Intramedullary spinal cord metastases: diagnosis and treatment - an illustrated review Acta Neurochir (Wien) 2004 146 12 1347 1354 10.1007/s00701-004-0386-1 15526223

[B14] Schiff D O'Neill BP Intramedullary spinal cord metastases: clinical features and treatment outcome Neurology 1996 47 4 906 912 8857717 10.1212/wnl.47.4.906

[B15] McCarron MO Flynn PA Pang KA Hawkins SA Traumatic Brown-Séquard-plus syndrome Arch Neurol 2001 58 9 1470 1472 10.1001/archneur.58.9.1470 11559320

[B16] Koehler PJ Endtz LJ The Brown-Séquard syndrome. True or False? Arch Neurol 1986 43 9 921 924 3741208 10.1001/archneur.1986.00520090051015

[B17] Metser U Lerman H Blank A Lievshitz G Bokstein F Even-Sapir E Malignant involvement of the spine: assessment by 18F-FDG PET/CT J Nucl Med 45 2 279 284 14960648

[B18] Schiff D Shaw EG Cascino TL Outcome after spinal re-irradiation for malignant epidural spinal cord compression Ann Neurol 1995 37 5 583 589 10.1002/ana.410370507 7755352

[B19] Choucair AK Myelopathies in the cancer patient: incidence, presentation, diagnosis and management Oncology 1991 5 7 25 31 1837475

[B20] Fornari M Pluchino F Solero CL Giombini S Luccarelli G Oliveri G Lasio G Microsurgical treatment of intramedullary spinal cord tumours Acta Neurochir Suppl (Wein) 1988 43 3 8 10.1007/978-3-7091-8978-8_1 3213654

